# 

**Published:** 1879-12-20

**Authors:** 


### EDITORIAL AND MISCELLANEOUS.

### *DR. POWELL'S INTRODUCTORY.*

We so far trespass upon our custom of publishing only short articles as to give place to the introductory delivered by our Senior Editor, Prof. Powell, at the opening of the *Southern Medical College* in this city. It is done by request, and in the hope that it may interest the profession and accomplish good by its eloquent disclosure of the noble and patriotic designs of the trustees in establishing the Institution. To inquiries as to the present status of the school, we are pleased to announce that it has now over fifty matriculates. An extraordinary opening, truly, for a new Institution. W.

### *EDITORIAL NOTICES.*

*Spectacles.*—If you want good spectacles go to Er Lawshe's, jeweler, 47 Whitehall street, Atlanta, Ga. We speak from experience in regard to his glasses. They are superior. The house of Lawshe has an established reputation in this city. He is a staunch, reliable and good business man.

*Buffalo Lithia Springs.*—Please read the two new advertisements of these wonderful waters commencing with the present issue of our journal.

*John H. Brand & Co*—sole manufacturers of the celebrated *Burrowe's Lexington Mustard.*—The testimonials in regard to the excellence of this mustard are numerous. We have tested a sample of it, and it is, indeed, very fine. See the advertisement.

*Pemberton, Pullum & Co.*—A change in the firm of Pemberton, Samuels & Reynolds will be perceived, Messrs. Samuels & Reynolds retiring, and Mr. Pullum taking their place. Mr. Pullum is an excellent man, and the firm will be among the very best in the Southern States. We can safely recommend this house to the patronage of our readers. See their advertisement.

*A. S. Barnes & Co., National Educational Publishers.*—This enterprising establishment has generously donated to the Southern Medical College Library the following works: Elements of Zoology, by Chambers; Physiology and Health, by Jarvis; Darby's Chemistry; Darby's Botany; Familiar Lessons in Botany; Elements of Physiology, by Hamilton; Lectures on Natural History, by Chadbourne.

*Generosity of D. Appleton & Co., New York.*—This splendid, wealthy and reliable house stands very high among American publishing establishments. They are noted for the extent and excellence of their publications, and for a benevolence and patriotism which embraces all sections of our common country. We are pleased to acknowledge the following works donated by this house to the *Southern Medical College Library*, at Atlanta, Ga.

Examination of Medicinal Chemicals; Flint's Text Book of Human Physiology; Neuralgia, etc., by Anstie; Treatment of Syphilis, by Keyes; Handbook of Uterine Therapeutics, by Tate; Practical Manual of the Treatment of Club Foot, by Sayer; General Convolutions of Man, by Ecker; Pulmonary Consumption, by Bennet; Orthopedic Surgery and Diseases of the Joints, by Sayre; Manual of Midwifery, by

Schroder; Hospital, History and Construction, by Wylie; Genito-Urinary Diseases, with Syphilis, by VanBuren & Keyes; Meteria Medica and Therapeutics, by Barthelow; Nenman's Handbook of Skin Diseases, by Bulkley; Niemyer's Text Book Practical Medicine, 2 Vols.; Compendium of Children's Diseases, by Steiner; Emergencies and How to Treat Them, by Howe; Management of Infancy, by Combs; Reparative Surgery, by Buck; Clinical Lectures on Diseases of the Nervous System, by Hammond; Obstetric Operations, by Barnes; The Puerperal Diseases, by Barker; Ovarian Tumors, by Peaslee; Diseases of Bones, by Markoe; Clinical Electro-Therapeutics, by Hamilton; Galvano Therapeutics, by Neftel; Diseases of the Ovaries, by Wells; Works of Sir J. Y. Simpson, 2 Vols.; Histology and Histo-Chemistry of Man, by Frey; Diseases of Children, by Vogel.

RECEIPTED.—1879—M. T. Anderson, W. S. Hays, A. Williamson, J. T. Cleveland, J. M. Mahan, W. H. Stewart. J. H. Horne; '79 and '80, A. H. Smith, H. J. Walker' Wm. M. Green, T. D. Hare; '80, J. T. Baker, H. Perdue, Robert Kelly, J. A. Holloway, J. W. Gilbert, J. H. Glover, C. F. Rogers, Eli Perkins, T. T. Christopher, A. L. Smoot, E. D. Simons, A. J. Graves, G. M. Pool, B. F. Stallions, J. J. Snell; '79 and '80, F. M. Thomason.

### TO OUR SUBSCRIBERS.

As this is the closing number of the present volume, we feel it appropriate to present to the many readers of THE RECORD our thanks for their patronage in the past, and our request for its continuance in the future.

Our acknowledgements are warmly tendered to all who have contributed to our pages, and who have aided in extending our circulation. We trust that they will continue to help us, and that we shall have the sympathy and co-operation of every subscriber in our efforts to advance the medical literature and to benefit the Profession in the Southern section of the Union.

We are pleased to state that our list is steadily growing—that the RECORD is more than self-sustaining, and that our plans for the future give promise of continued improvement and of increased advantages to our readers.

Renewals are rapidly coming in, giving evidence of continued confidence in the journal, and accompanied in many instances with very complimentary expressions in regard to its excellencies. The following extract is from one of recent date (December 10th), by Dr. W. C. Moore, of Clarkston, Ga. :

"I will say for the RECORD that its entire plan and get up is most admirably conceived for the busy practitioner. It is just the right size. I have tried the smaller and cheaper journals; they are not satisfactory, while the larger ones cost more and contain too much useless detail. Yours is compact, plain, varied and practical. Go ahead on the same line; you are doing a good work, and the Profession will surely sustain you."

Under these circumstances we take for granted that all our subscribers desire their journal continued for 1880, and shall so enter them unless notified to the contrary before the issue of the 20th of January next.

Our subscribers may obtain club rates on renewal by sending one name with their own, or $3.50 for the two.

We are very thankful to those who, mindful of our necessities, have promptly remitted their subscriptions. Many, who were granted short indulgence, have not responded, subjecting us to serious annoyance and inconvenience.

We earnestly request that all who are in arrears will remit at once. The demands of the closing year and its increased expenses are upon us, and we trust that remittances both of back dues and renewals wlll be made without delay. Remember the demands upon us are cash, and when you cripple a Journalist, thousands will feel it. Of your creditors, therefore, give us the precedence.

And now, friends, as we labor for the Profession, will you not extend a helping hand? How easy it would be for each of our readers to procure us one subscriber and thus double our list.

In conclusion, accept our salutations and greetings for a happy new year! THE EDITORS.

**N. B Bound volumes of the RECORD we can furnish at $2.50 and postage; or parties can have their binding done here at $1.00 per volume. The index of volume for 1880 contains more items than any monthly medical journal in the United States.**

